# Cost-effectiveness analysis of Enfortumab vedotin and pembrolizumab for advanced urothelial carcinoma

**DOI:** 10.1097/MD.0000000000048312

**Published:** 2026-04-10

**Authors:** Hao Cheng, Jun Li, Ningying Mao

**Affiliations:** a School of International Pharmaceutical Business, China Pharmaceutical University, Nanjing, China.

**Keywords:** advanced urothelial carcinoma, cost-effectiveness analysis, Enfortumab vedotin and pembrolizumab, price simulation, US

## Abstract

**Background:**

To evaluate the cost-effectiveness of Enfortumab vedotin and pembrolizumab (EVP) as a new first-line treatment option for advanced urothelial carcinoma compared to standard chemotherapy (gemcitabine with cisplatin or carboplatin) from the perspective of the US healthcare payer.

**Methods:**

A partitioned survival model was used to evaluate the cost-effectiveness of EVP compared to standard chemotherapy, incorporating 3 health states: progression-free survival (PFS), progressive disease, and death. The analysis was conducted from the perspective of a US healthcare payer (Veterans Affairs, VA) using a 21-day simulation cycle over a 30-year horizon. Survival data were derived from the EV-302 trial and reconstructed using standard parametric models as well as flexible parametric models. Costs and utility values were sourced from public databases and literature. Sensitivity and scenario analyses assessed the impact of key parameters and different subgroups, with price simulations identifying the threshold price for cost-effectiveness. Total costs (in US dollars), life years, quality-adjusted life years (QALYs), and incremental cost-effectiveness ratios were used as main outcomes.

**Results:**

In the base-case analysis, the lifetime cost of EVP was $989,917, providing an effectiveness of 2.50 QALYs and 4.86 life years. In contrast, chemotherapy cost $166,397 with an effectiveness of 1.49 QALYs and 2.83 life years. The incremental cost-effectiveness ratio for EVP compared to chemotherapy was $814,875 per QALY, indicating it is not cost-effective at the $150,000 threshold. Sensitivity analyses highlighted that the utility for PFS and the pricing of Enfortumab vedotin (EV) significantly impacted cost-effectiveness. Probabilistic sensitivity analysis confirmed chemotherapy as the most cost-effective option across a willingness-to-pay range of $0 to $300,000. Price simulations suggested that EVP would become cost-effective compared to chemotherapy if the price of EV were reduced to $593 per 20 mg. Subgroup analysis showed that at its original price, EV was not cost-effective in any subgroup. However, reducing its price to 22 to 29% of the original made EVP economically viable for various subgroups.

**Conclusions:**

EVP is more effective than chemotherapy but not cost-effective from the perspective of the VA. To achieve cost-effectiveness, a price reduction for EV is necessary.

## 1. Introduction

In the United States, advanced urothelial carcinoma (aUC) is a prevalent cancer of the urinary system. The National Cancer Institute reported over 79,000 new cases and more than 16,000 deaths in 2017.^[1]^ Globally, it causes over 165,000 deaths each year, making it the ninth most common cancer. By 2020, deaths reached 179,000. Approximately 17% of patients are diagnosed with metastatic disease, with a 5-year survival rate of only 11.7%.^[2]^ The economic impact is substantial, as it ranks among the costliest cancers to manage, with lifetime treatment expenses per patient estimated between $117,000 and $191,000, varying by diagnosis stage.^[3]^ Urothelial carcinoma, making up about 90% of bladder cancer cases, is the most common histological type.^[4]^

For aUC, platinum-based chemotherapy has been the main treatment for years, but with limited success and low long-term survival rates.^[5]^ Maintenance therapy with avelumab can extend survival, while many patients don’t receive it due to disease progression or death.^[6]^ Nivolumab combined with gemcitabine-cisplatin has shown slight survival improvements, but other trials with chemo and immune therapies haven’t significantly increased survival.^[7]^ Enfortumab vedotin and pembrolizumab (EVP) have each improved survival in previously treated patients. Preclinical studies suggest that combining these 2 enhances antitumor effects.^[8–10]^ This combination has received accelerated approval in the U.S. for patients who can’t undergo cisplatin-based chemotherapy, based on high response rates in early studies.^[11]^

Enfortumab vedotin (EV) is an antibody-drug conjugate targeting nectin-4, and pembrolizumab is a PD-1 inhibitor. In the EV-302 phase 3 trial, patients with untreated aUC were randomly assigned to either EV-pembrolizumab or platinum-based chemotherapy.^[11]^ With 886 participants, the trial showed that those receiving EVP experienced longer progression-free survival (PFS, median of 12.5 months) compared to chemotherapy (6.3 months). Overall survival (OS) was also greater (31.5 months vs 16.1 months). Severe adverse events (SAEs) occurred in 55.9% of the combination group versus 69.5% in the chemotherapy group.^[11]^

While EVP are effective and safe for aUC, it’s crucial to weigh their clinical benefits against the high costs, particularly given the expense of new treatments. The aim is to evaluate the cost-effectiveness of EVP compared to platinum-based chemotherapy, from the perspective of US healthcare payers.

## 2. Method

### 2.1. Model overview

**Figure 1.**
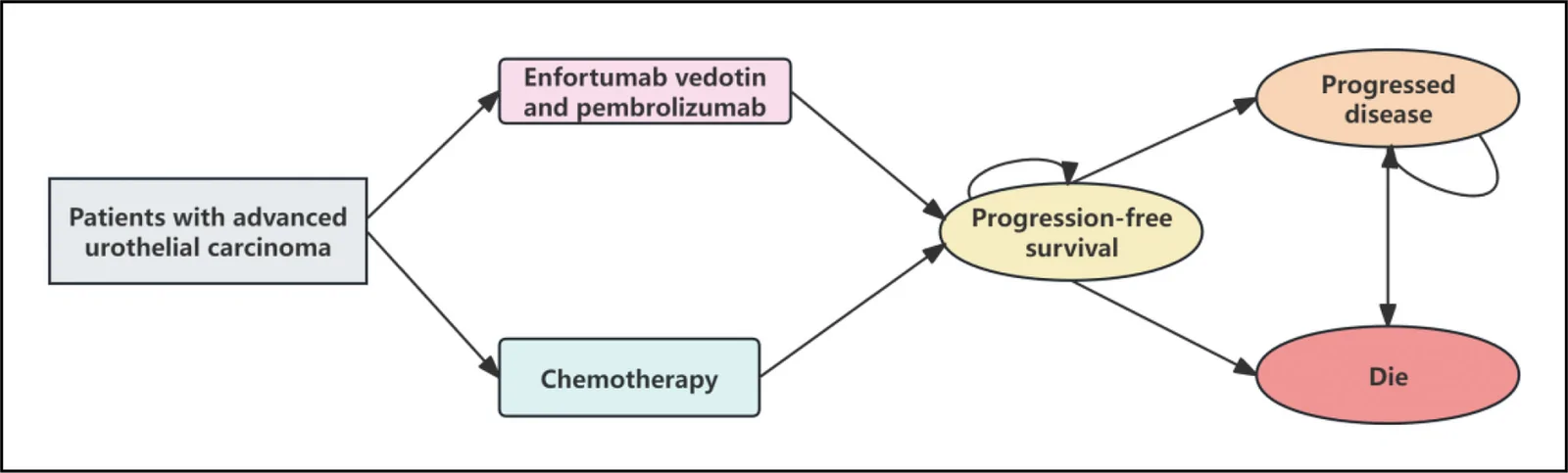
Model structure plot.

Using methods from previous economic evaluations for renal cell carcinoma treatment submitted to the Scottish Medicines Consortium and the National Institute for Health and Care Excellence (NICE), a partitioned survival model (PSM) with 3 health states (Fig. 1) – PFS, progressive disease (PD), and death – was developed to evaluate the incremental cost-effectiveness ratio (ICER) of EVP compared to standard chemotherapy (gemcitabine and either cisplatin or carboplatin).^[12]^ The model was analyzed from the viewpoint of a US healthcare payer (Veterans Affairs, VA), using a 21-day simulation cycle and a 30-year time horizon to ensure all patients eventually reach the state of death. Both costs and outcomes were discounted at 3%.^[13]^ The willingness-to-pay (WTP) threshold per QALY was established at $150,000, with a range between $100,000 and $200,000.^[14]^ For further details, see Table 1.

**Table 1:** Model parameters.

Parameters	Value (mean, uncertainty interval)	Distribution	Source
Cost ($)			
Cost_best supportive care/per cycle	1213.00 (937.00–1438.00)	Gamma	^[15]^
Cost_end-of-life care	11,939.77 (9551.82–14,327.72)	Gamma	^[16]^
Disease management costs/per cycle	567.65 (454.12–681.17)	Gamma	^[17]^
Administration cost per included treatment/per cycle	132.16 (105.73–158.59)	Gamma	^[17]^
Monitoring costs per included treatment/per cycle	44.72 (35.78–53.67)	Gamma	^[17]^
Price of Enfortumab vedotin per 20 mg	2576.58 (2061.26–3091.90)	Gamma	^[18]^
Price of pembrolizumab per 100 mg	5317.27 (4253.82–6380.72)	Gamma	^[18]^
Price of gemcitabine per 2000 mg	28.14 (22.51–33.77)	Gamma	^[18]^
Price of gemcitabine per 200 mg	4.02 (3.22–4.82)	Gamma	^[18]^
Price of cisplatin per 50 mg	6.93 (5.54–8.32)	Gamma	^[18]^
Price of cisplatin per 100 mg	13.60 (10.88–16.32)	Gamma	^[18]^
Price of carboplatin per 50 mg	8.20 (6.56–9.84)	Gamma	^[18]^
Price of carboplatin per 150 mg	20.95 (16.76–25.14)	Gamma	^[18]^
Cost of peripheral sensory neuropathy	7487.00 (5989.60–8984.40)	Gamma	^[19]^
Cost of maculopapular rash	15,709.00 (12,567.20–18,850.80)	Gamma	^[15]^
Cost of fatigue	4261.30 (3409.04–5113.56)	Beta	^[20]^
Cost of diarrhea	4163.50 (3330.80–4996.20)	Beta	^[20]^
Cost of anemia	4368.00 (3494.40–5241.60)	Beta	^[21]^
Cost of hyperglycemia	17,621.42 (14,097.14–21,145.70)	Beta	^[22]^
Cost of neutropenia	5937.00 (4749.60–7124.40)	Beta	^[21]^
Cost of neutrophil count decreased	5937.00 (4749.60–7124.40)	Beta	^[21]^
Cost of thrombocytopenia	4014.00 (3211.20–4816.80)	Beta	^[21]^
Cost of platelet count decreased	4014.00 (3211.20–4816.80)	Beta	^[21]^
Utility and disutility			
Grade 1–2 AE disutility	0.01 (0.01–0.02)	Beta	^[15]^
Grade 3+ AE disutility	0.28 (0.21–0.35)	Beta	^[15]^
Utility for PFS	0.84 (0.67–1.00)	Beta	^[21]^
Utility for PD	0.80 (0.64–0.96)	Beta	^[21]^
Incidence of adverse event			
Overall SAE_EVP	0.56 (0.45–0.67)	Beta	^[11]^
Overall SAE_chemotherapy	0.70 (0.56–0.83)	Beta	^[11]^
Overall any AE_EVP	0.97 (0.94–1.00)	Beta	^[11]^
Overall any AE_chemotherapy	0.96 (0.92–1.00)	Beta	^[11]^
Peripheral sensory neuropathy_EVP	0.04 (0.03–0.04)	Beta	^[11]^
Maculopapular Rash_EVP	0.08 (0.06–0.09)	Beta	^[11]^
Fatigue_EVP	0.03 (0.02–0.04)	Beta	^[11]^
Diarrhea_EVP	0.04 (0.03–0.04)	Beta	^[11]^
Anemia_EVP	0.03 (0.03–0.04)	Beta	^[11]^
Hyperglycemia_EVP	0.05 (0.04–0.06)	Beta	^[11]^
Neutropenia_EVP	0.05 (0.04–0.06)	Beta	^[11]^
Neutrophil count decreased_EVP	0.03 (0.02–0.03)	Beta	^[11]^
Thrombocytopenia_EVP	0.01 (0.00–0.01)	Beta	^[11]^
Fatigue_chemotherapy	0.04 (0.03–0.05)	Beta	^[11]^
Diarrhea_chemotherapy	0.01 (0.01–0.01)	Beta	^[11]^
Anemia_chemotherapy	0.31 (0.25–0.38)	Beta	^[11]^
Neutropenia_chemotherapy	0.30 (0.24–0.36)	Beta	^[11]^
Neutrophil count decreased_chemotherapy	0.09 (0.07–0.11)	Beta	^[11]^
Thrombocytopenia_chemotherapy	0.19 (0.16–0.23)	Beta	^[11]^
Platelet count decreased_chemotherapy	0.07 (0.05–0.08)	Beta	^[11]^
Subsequent active treatment			
Subsequent active treatment_EVP	0.32 (0.25–0.38)	Beta	^[11]^
Subsequent active treatment_chemotherapy	0.71 (0.56–0.85)	Beta	^[11]^
Others			
Body weight/kg	71.40 (64.26–78.54)	Normal	^[23]^
Body surface area/m^2^	1.80 (1.62–1.98)	Normal	^[24]^
Creatinine clearance (mL/min)	65.80 (59.22–72.38)	Normal	^[24]^
Discount	0.03 (0.00–0.08)	Beta	^[25]^

AE = adverse event, EVP = Enfortumab vedotin and pembrolizumab, PD = progressed disease, PFS = progression-free survival, SAE = severe (grade 3+) adverse event.

### 2.2. Survival estimates

The PSM estimated health state occupancy using the area-under-the-curve method from OS and PFS curves. Estimated survival for EVP, or chemotherapy, were derived from the EV-302 trial results. Data points were extracted using GetData Graph Digitizer and individual patient data (IPD) were reconstructed with Guyot’s method.^[26]^ Validation of the reconstructed IPD accuracy is provided in Methods S1 (Supplemental Digital Content, https://links.lww.com/MD/R673). Reconstructed IPD were parameterized using various models, including standard parametric models, Royston–Parmar (RP) spline models, fractional polynomial models, and restricted cubic spline models.^[27]^ Goodness-of-fit was assessed by examining survival curves visually and applying the Akaike information criterion. A model with a lower Akaike information criterion and a strong visual alignment indicated superior performance. In addition, the clinical plausibility of long-term extrapolations was reviewed for face validity based on clinical expert judgment. Survival modeling utilized R.^[27]^ Ultimately, the RP model was chosen for the OS curve in the chemotherapy group, while the log-logistic model was selected for the EVP group. RP models were also used for the PFS curves in both groups. More details on model selection, along with visual inspection results, can be found in Tables S1–S4 (Supplemental Digital Content, https://links.lww.com/MD/R674), Figures S1–S4 (Supplemental Digital Content, https://links.lww.com/MD/R675).

### 2.3. Cost and utility estimates

This study focuses solely on direct medical costs. Medication unit prices were derived from the Federal Supply Schedule average sales prices. The Federal Supply Schedule provides negotiated prices between pharmaceutical companies and the VA, a major medication purchaser in the US.^[18]^ According to the EV-302 trial, in the base-case analysis of this study, it was assumed that 54% of patients received cisplatin. Subsequent therapies were modeled in terms of costs after progression; no additional independent survival benefit was assigned to subsequent therapies because OS/PFS were informed by EV‑302 trial curves, thereby avoiding double counting (Fig. S5, Supplemental Digital Content, https://links.lww.com/MD/R675). 31.7% of patients in the EVP group received active treatment (platinum-based monotherapy) after progression, while 70.5% of patients in the chemotherapy group continued with active treatment (primarily with immune checkpoint inhibitors). The study considered grade 3+ SAEs occurring in over 3% of EV-302 trial patients in the EVP or chemotherapy groups. Treatment costs for these SAEs were obtained from Nationwide Healthcare Cost and Utilization Project databases and published cost-effectiveness analyses focused on aUC.^[20]^ We referred to evidence in the urologic‑oncology literature on urine‑based molecular follow-up strategies and their diagnostic performance to support cost quantification in the model.^[28–36]^ Costs for disease management costs, administration and monitoring costs, best supportive care, and end-of-life care were drawn from Centers for Medicaid and Medicare Services and existing literature.^[15–17]^ To calculate chemotherapy costs, it was assumed that the adult weight is 71.4 kg, the body surface area is 1.8 square meters, and the creatinine clearance is 65.8 mL/minute.^[23,24]^ All costs were adjusted to 2023 US dollars using the Personal Consumption Expenditures Price Index for healthcare services.^[37]^ Details are in Table 1, with the cost and resource utilization breakdown in Tables S5–S7 (Supplemental Digital Content, https://links.lww.com/MD/R674).

The EuroQol-5 Dimensions instrument provided utility values for various health states in this study related to quality of life. Because EV-302 did not report health-state utility data, we used utility inputs from external study; a description of the comparability between the 2 study populations is provided in Method S2 (Supplemental Digital Content, https://links.lww.com/MD/R673). Each health state was assigned a quality-of-life weight, with utility scores ranging from 1 (full health) to 0 (death), based on previous research.^[38]^ The utility scores were 0.842 for the PFS state and 0.800 for the PD state. We accounted for utility loss from adverse events, using disutility values from similar aUC studies, assuming a one-cycle duration. Specifically, the disutility for grade 1 to 2 AEs was 0.01, and for grade 3 or higher AEs, it was 0.28.^[15]^ More details are provided in Table 1.

### 2.4. Discounting scenario and uncertainty analyses

To address policy-relevant uncertainty related to confidential rebates/discounting in oncology, we conducted a price-threshold analysis for EV. EV prices were tested from zero to full discount in 1% steps to identify the cost-effective threshold. Cost-effectiveness was measured by the incremental net monetary benefit (INMB), where a value above 0 indicates the treatment is cost-effective.^[39]^

Deterministic sensitivity analysis (DSA) assessed how key parameters affect the ICER, using tornado diagrams for results. Parameters varied within literature ranges or ±20% of baseline if limits were unknown. Probabilistic sensitivity analysis (PSA) involved 10,000 Monte Carlo simulations. Costs followed a gamma distribution, while beta distribution was used for probabilities, proportions, and utilities. Cost-effectiveness acceptability curves analyzed probabilities over a $0 to $500,000 WTP range. Table 1 outlines the uncertainty sources.

We performed scenario analysis (Scenario 1) to tackle uncertainties in survival estimates, utilizing hazard ratio values from a Cox proportional hazards model based on the North American patient population to determine clinical transition probabilities. We additionally conducted a structural scenario analysis (Scenario 2) assuming prolonged post-progression active treatment duration (i.e., active treatment was assumed to continue until 6 months before death) to assess the impact of alternative, conservative subsequent-therapy duration assumptions on the ICER.

We conducted separate economic evaluations for the provided subgroups (age [<65 and ≥65], gender [male vs female], ECOG status [0 vs 1/2], primary disease site [upper vs lower tract], liver metastases [present vs absent], PD-L1 expression [low vs high], cisplatin eligibility, metastasis site [visceral vs lymph node only], and renal function [normal vs mild vs moderate/severe impairment]) to assess the cost-effectiveness of EVP compared to chemotherapy, including price simulations.

## 3. Results

In the base-case analysis, the cost of EVP was $989,917 with an effectiveness of 2.50 QALYs and 4.86 life years. Chemotherapy cost $166,397, with an effectiveness of 1.49 QALYs and 2.83 life years. The cost difference for EVP compared to chemotherapy was $823,520, with an effectiveness difference of 1.011 QALYs, resulting in an ICER of $814,875/QALY. This is significantly above the $150,000 WTP threshold, indicating that EVP is not cost-effective compared to chemotherapy.

**Figure 2.**
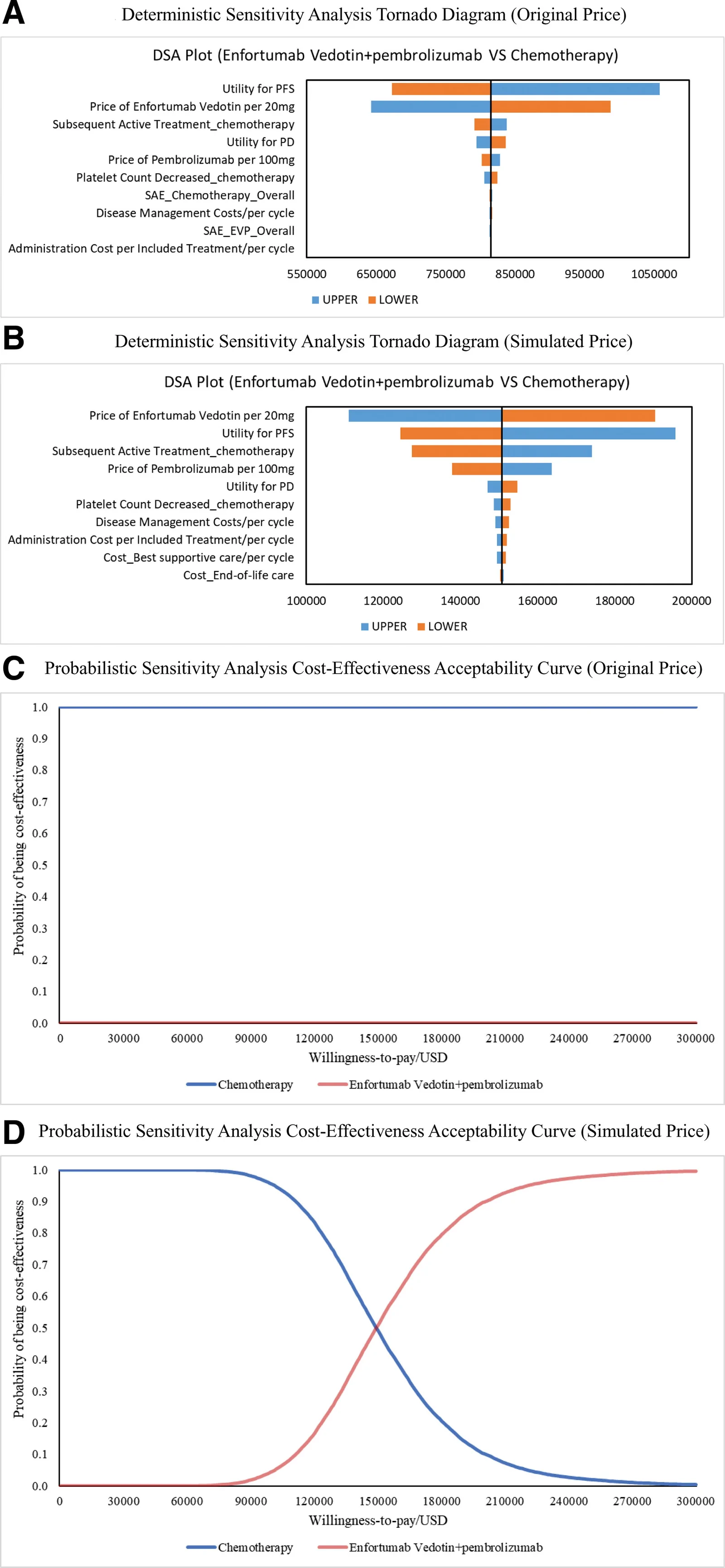
Sensitivity analysis results. PD = progressed disease, PFS = progression-free survival, SAE = severe adverse event.

DSA results indicated that the utility for PFS and the price of EV had significant impacts on cost-effectiveness. Subsequent chemotherapy and utility for PD also had some influence, but to a lesser extent. The costs of pembrolizumab, disease management, and administration were less impactful. Results also showed upper and lower ranges for each factor, highlighting variability in their effects. Even with fluctuations within their upper and lower limits, the EVP was not cost-effective compared to chemotherapy, as ICER values consistently exceeded the WTP threshold. More details are provided in Figure 2A.

PSA results further confirmed the robustness of the base-case analysis conclusions. According to the cost-effectiveness acceptability curves, across a WTP threshold from $0 to $300,000, chemotherapy consistently showed the highest likelihood of being the most cost-effective choice. Specifically, within this study’s threshold range, the probability of chemotherapy being more cost-effective compared to EVP was nearly 100%. See Figure 2B for more details.

### 3.1. Scenario analysis

In Scenario 1, treatment comparison showed that chemotherapy costs $166,397 with an effectiveness of 1.49 QALYs and a life-year of 2.83. In contrast, the EVP costs $989,917 with an effectiveness of 1.98 QALYs and a life-year of 3.80. The difference in cost between EVP and chemotherapy is $617,648, with an additional effectiveness of 0.493 QALYs, resulting in an ICER of $1252,248 per QALY for EVP compared to chemotherapy.

In Scenario 2, treatment comparison showed that chemotherapy costs $156,376 with an effectiveness of 1.49 QALYs and 2.83 life-years. In contrast, EVP costs $976,886 with an effectiveness of 2.50 QALYs and 4.86 life-years. The incremental cost of EVP versus chemotherapy is $820,510, with an additional 1.01 QALYs, resulting in an ICER of $811,897 per QALY for EVP compared to chemotherapy. More details are provided in Table 2.

**Table 2:** Summary of base-case analysis and scenario analysis results.

Treatment	Cost/$	Effectiveness/QALY	Life-year (yr)	Chemotherapy vs EVP		
				Differences in cost/$	Differences in effectiveness/QALY	ICER ($/QALY)
Base-case analysis						
Chemotherapy	166,397	1.49	2.83	–	–	–
EVP	989,917	2.50	4.86	823,520	1.01	814,875
Scenario analysis 1						
Chemotherapy	166,397	1.49	2.83	–	–	–
EVP	784,044	1.98	3.80	617,648	0.493	1,252,248
Scenario analysis 2						
Chemotherapy	156,376	1.49	2.83	–	–	–
EVP	976,886	2.50	4.86	820,510	1.01	811,897

EVP = Enfortumab vedotin and pembrolizumab, QALY = quality-adjusted life years.

### 3.2. Price simulation and subgroup analysis

**Figure 3.**
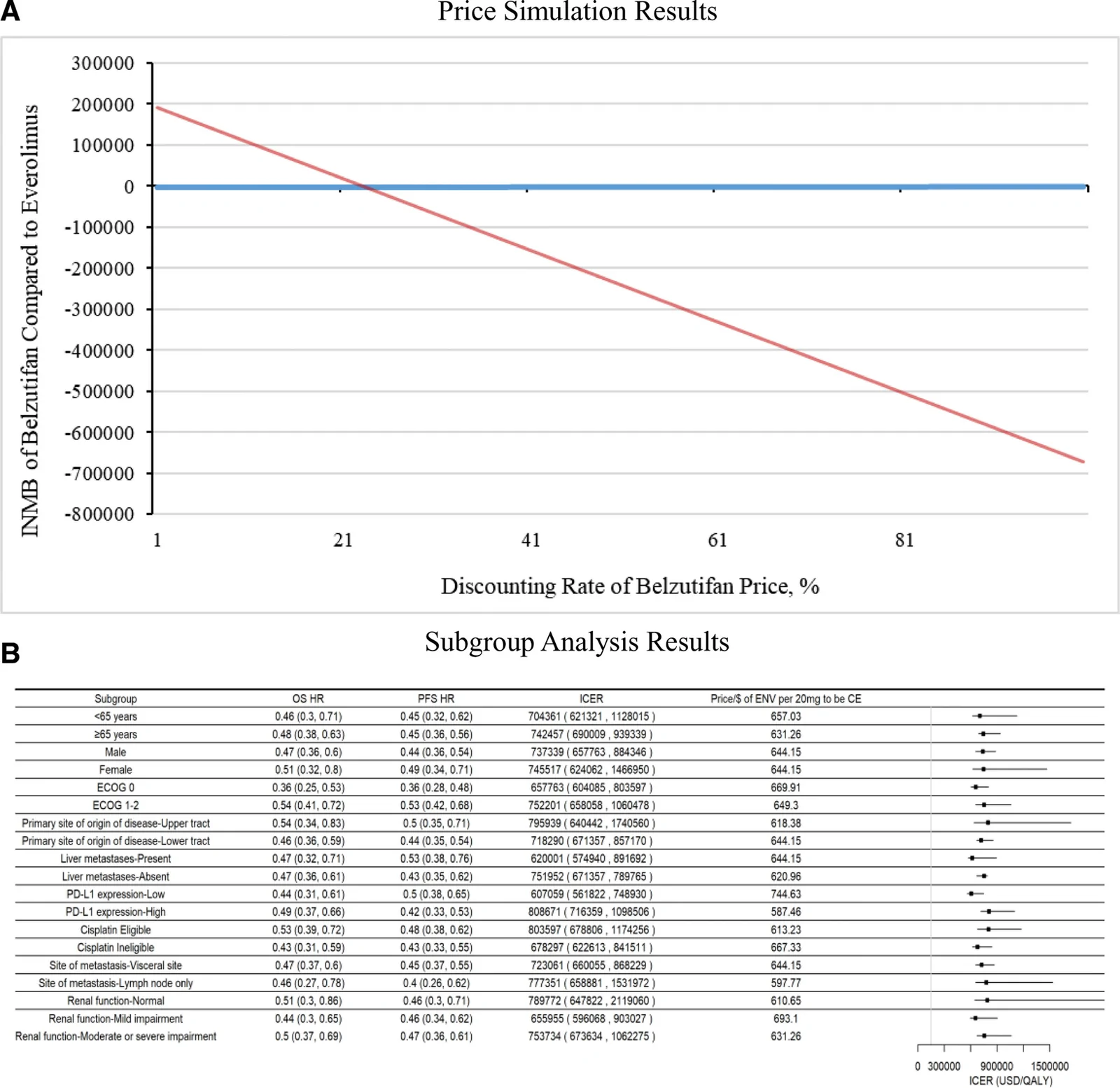
Price simulation and subgroup analysis results. CE = cost-effective, HR = hazards ratio, ICER = incremental cost-effectiveness ratio, INMB = incremental net monetary benefit, OS = overall survival, PFS = progression-free survival.

In the price simulation, the INMB of EVP compared to chemotherapy increased as the discount rate increased. EV became cost-effective when its price dropped to 23% of the original, amounting to $593 per 20 mg. Figure 3A illustrates that as the discount rate rose from 0.01 to 0.99, the INMB increased from around −663,213 to approximately 190,979. At this price, the DSA results (Fig. 2C) indicate that drug price, subsequent treatment options, and utility values are the main factors affecting cost-effectiveness. The PSA results (Fig. 2D) further confirm that, at this price, the probability of EVP being cost-effective is approximately 50% when the WTP threshold is $150,000, and the cost-effectiveness of EVP improves as the threshold increases.

In the subgroup analysis, the ICER for patients under 65 is $704,361 at a price of $657.03. In the ≥65 group, the ICER is $742,457, with a price of $631.26. Males have an ICER of $737,339, priced at $644.15, while females have an ICER of $745,517, priced at the same rate. ECOG 0 shows an ICER of $657,763, priced at $669.91, and ECOG 1 to 2 has an ICER of $752,201, priced at $649.3. Upper tract origin has an ICER of $795,939, priced at $618.38, while lower tract origin is $718,290, priced at $644.15. For patients with liver metastases, the ICER is $620,001 at a price of $644.15, while for those without liver metastases, the ICER is $751,952 at a price of $620.96. Low PD-L1 expression patients has an ICER of $607,950, priced at $744.63, whereas high expression has an ICER of $808,671, priced at $587.46. Cisplatin eligible individuals have an ICER of $803,597, priced at $613.23, while ineligible patients have an ICER of $678,297, priced at $667.33. Visceral site metastasis shows an ICER of $723,061, priced at $644.15, while lymph node-only metastasis has an ICER of $777,731, priced at $597.77. Normal renal function has an ICER of $789,772, priced at $610.65; mild impairment has an ICER of $655,955, priced at $693.1; and moderate to severe impairment has an ICER of $753,734, priced at $631.26. For more details, see Figure 3B. Sample size, number of events for each subgroup are provided in Figures S6 and S7 (Supplemental Digital Content, https://links.lww.com/MD/R675). Subgroup analyses should be interpreted with caution. As several subgroups include comparatively small numbers of patients and events (e.g., the liver metastases subgroup and the moderate/severe renal impairment subgroup). Accordingly, the subgroup-specific treatment-effect estimates and ICER results are considered exploratory and are subject to increased statistical uncertainty.

### 3.3. Model validation

We conducted model validation to improve credibility in 3 aspects. Face validity was assessed through clinical expert review to confirm the clinical plausibility of the mode. Internal validity was assessed by reconstructing IPD from EV‑302 and reproducing Kaplan–Meier curves within the observed trial horizon; reconstructed outcomes closely matched published trial anchors (Methods S1, Supplemental Digital Content, https://links.lww.com/MD/R673). External consistency was assessed by comparing our effectiveness results with a published EVP economic model using the same pivotal evidence^[40]^; QALYs were comparable (published study: chemotherapy 1.49, EVP 2.50; our study: chemotherapy 1.33, EVP 2.45).

## 4. Discussion

In the analysis, EVP costs $989,917 with an effectiveness of 2.50 QALYs, while chemotherapy costs $166,397 with 1.49 QALYs. The ICER for EVP is $814,875/QALY, which exceeds the $150,000 threshold, indicating it is not cost-effective. Sensitivity analyses showed significant impacts from progression-free survival utility and EVP pricing, but EVP was consistently not cost-effective across variable ranges. Probabilistic analysis confirmed that chemotherapy was almost always more cost-effective within a WTP threshold of $300,000. Scenario analysis showed an ICER of $1252,248/QALY for EVP, and price simulations indicated that EVP became cost-effective when its price was reduced to 23% of EV’s original price. Subgroup analysis revealed high incremental cost-effectiveness ratios (ICERs) across various groups, including age, gender, and health conditions, with no subgroup showing EVP as cost-effective, highlighting that price reductions improved cost-effectiveness, but ICERs remained above acceptable thresholds.

Several articles have evaluated the cost-effectiveness of systemic therapies for aUC patients. Lin et al’s study^[41]^ evaluated the cost-effectiveness of avelumab maintenance therapy for aUC. Using data from a clinical trial, the analysis found that avelumab increases QALYs but results in high ICERs of $699,065/QALY for the overall population and $521,850/QALY for PD-L1-positive patients. Both exceed the $200,000/QALY threshold, indicating avelumab is not cost-effective from a US payer perspective. Xie et al’s study^[42]^ assessed the cost-effectiveness of avelumab maintenance therapy for advanced urothelial cancer from the US and China payer perspectives. Using a Markov simulation model based on the JAVELIN Bladder 100 trial, the ICER for avelumab was $38,369.50 per QALY in the overall population and $16,150.29 in the PD-L1-positive population. However, compared to best supportive care, the ICERs were $241,610.25 and $100,528.29 per QALY, respectively. Avelumab was deemed cost-effective in both populations in the US at a $150,000 WTP threshold, but not in China at a $30,447.09 threshold. Qin et al^[43]^ evaluated the cost-effectiveness of atezolizumab plus platinum-based chemotherapy for metastatic urothelial cancer from the US payer perspective. The Markov model showed atezolizumab provided an additional 0.39 QALYs at an incremental cost of $170,759 per QALY compared to the placebo group. The overall ICER was $434,317 per QALY, indicating it is not cost-effective at the $150,000 WTP threshold. Subgroup analysis showed an ICER of $325,236 per QALY for patients with at least 5% PD-L1 expression. Sensitivity analyses highlighted sensitivity to drug costs and PFS. Hale et al^[44]^ assessed pembrolizumab’s cost-effectiveness as a first-line treatment for cisplatin-ineligible patients with PD-L1-positive aUC. Using a PSM, the analysis compared pembrolizumab to carboplatin plus gemcitabine. Over 20 years, pembrolizumab showed a gain of 2.58 life-years and 2.01 QALYs, with additional costs of $158,561. The incremental cost-effectiveness ratio was $78,925 per QALY. Li’s study^[19]^ assessed the cost-effectiveness of EV plus pembrolizumab versus chemotherapy for metastatic urothelial carcinoma from the Medicare perspective and concluded it was not cost-effective; in contrast, our study adopted the VA perspective, utilized a more flexible model, and conducted additional analyses, including subgroup and pricing evaluations. However, ICERs reported across published studies should not be interpreted as a head‑to‑head economic ranking of first‑line aUC options, because studies differ in perspective (e.g., VA vs Medicare/commercial), time horizon, survival‑extrapolation approach, costing year, and – most importantly – assumptions on post‑progression treatment and resource use. We therefore did not conduct a network meta-analysis to position EVP against all immunotherapy‑based combinations. A robust network meta-analysis would require a well‑connected evidence network and consistent endpoint definitions and follow‑up across trials, and would need to address cross‑trial heterogeneity (e.g., subsequent therapy patterns and maintenance use), which could introduce additional uncertainty and potentially bias comparative estimates.^[21,22,25]^ Accordingly, our conclusions focus on the within‑trial comparison supported by EV‑302.

This study is the first to assess the economic value of EVP in aUC using a flexible survival analysis model and conducted multiple uncertainty analyses to strengthen our conclusions. We also performed separate cost-effectiveness analyses for 19 subgroups, which is crucial for optimizing drug choices in precision medicine. Finally, we provided an evidence-based cost-effective price for EV, providing valuable insights for healthcare decision-makers, patients, and clinicians.

This study has several limitations. First, IPD from EV‑302 were not available, which restricted more granular subgroup analyses. Second, due to reporting constraints in EV‑302, the populations informing survival estimation and health‑state utilities were not identical, which may introduce additional uncertainty. Third, while EV‑302 provides direct randomized evidence versus platinum‑based chemotherapy, the lack of head‑to‑head RCT evidence versus other first‑line immunotherapy‑based combinations limited our ability to compare EVP with alternative active regimens beyond chemotherapy. Structural uncertainty also remains: post‑progression treatment patterns may vary across practice settings, and the model may not fully capture alternative subsequent treatment sequences. In addition, cumulative costs and outcomes associated with long‑term or late‑onset toxicities may not be fully reflected. Finally, we did not conduct a network meta‑analysis across immunotherapy‑based combinations; therefore, our results should not be interpreted as a formal economic ranking of EVP against all first‑line options. Future studies should validate key assumptions using real‑world data on post‑progression management, resource use, and long‑term toxicity.

## 5. Conclusion

EVP is more effective but not cost-effective compared to chemotherapy, as shown by sensitivity and scenario analyses due to its high costs relative to its benefits. For EVP to be cost-effective, its price needs to be reduced to 22 to 29% of the current price across different subgroups.

## Author contributions

**Conceptualization:** Ningying Mao, Hao Cheng.

**Data curation:** Hao Cheng.

**Formal analysis:** Hao Cheng.

**Funding acquisition:** Ningying Mao.

**Investigation:** Hao Cheng.

**Methodology:** Ningying Mao, Hao Cheng.

**Supervision:** Ningying Mao, Jun Li.

**Validation:** Ningying Mao.

**Writing – original draft:** Hao Cheng.

**Writing – review & editing:** Ningying Mao, Jun Li.

## Supplementary Material






