# Supplementary material for: Cost-effectiveness analysis of Enfortumab vedotin and pembrolizumab for advanced urothelial carcinoma
Source: Medicine (Baltimore). 2026 Apr 10;105(15):e48312. doi: 10.1097/MD.0000000000048312 (PMC13593259; doi:10.1097/MD.0000000000048312)
Supplement: Supplementary file 1 [file medi-105-e48312-s001.pdf]

Method S1 Reconstruction of individual patient data and validation against published EV-302 results

Because individual patient data (IPD) from EV-302 were not publicly available, we reconstructed pseudo-IPD from the published Kaplan–Meier (KM) curves using the method of Guyot et al. Briefly, the KM curves were digitized, and the published numbers at risk (when available) and total events were used to iteratively estimate individual event/censoring times that reproduce the reported KM step function.

To evaluate reconstruction accuracy and to characterize potential reconstruction-related bias, we performed a consistency check using key trial-reported "anchor" outcomes from the EV-302 publication. Specifically, we compared reconstructed versus published median progression-free survival (PFS), median overall survival (OS), and Cox proportional-hazards estimates (hazard ratios) for PFS and OS.

The reconstructed results closely reproduced the published EV-302 outcomes: reconstructed PFS hazard ratio 0.4618 versus published 0.45; reconstructed OS hazard ratio 0.4671 versus published 0.47. Reconstructed median PFS was 6.3 months in the chemotherapy arm and 12.6 months in the experimental arm, compared with published medians of 6.3 and 12.5 months, respectively. Reconstructed median OS was 16.2 months in the chemotherapy arm and 31.6 months in the experimental arm, compared with published medians of 16.1 and 31.5 months, respectively. Overall, these comparisons suggest that any bias introduced by Guyot reconstruction is limited for the key parameters used in the model.

Method S2 Utility source and population comparability

Trial-based health-state utility values were not reported in the EV-302 publication; therefore, utility inputs were sourced from an external first-line urothelial cancer study (Patterson et al., 2019). Although the utility inputs were obtained from Patterson et al., 2019 rather than EV-302, the two populations are comparable in several key, reportable baseline features. For example, the proportion of male patients is similar (EV-302: 77.8% in the enfortumab vedotin–pembrolizumab group and 75.7% in the chemotherapy group; Patterson et al., 2019: 77%), and the prevalence of liver metastases is also similar (EV-302: 22.6% and 22.3%, respectively; Patterson et al., 2019: 21%). Both studies represent the same indication and line of therapy (previously untreated locally advanced/unresectable or metastatic urothelial carcinoma). The median age differed modestly (EV-302: 69 years; Patterson et al., 2019: 74 years), but both cohorts predominantly comprised older patients. Detailed comorbidity indices (e.g., Charlson comorbidity index) were not reported in the publications and therefore could not be formally compared; however, both studies included clinically relevant comorbidity-related features (e.g., renal dysfunction/cisplatin ineligibility criteria), supporting partial comparability of the underlying patient health status. In addition, both studies were international multicenter trials that included substantial enrollment from North America and Europe, supporting geographic comparability.

To support replication, we further clarified how utilities and disutilities were implemented. Because EV-302 did not report preference-based utilities, all health-state utilities in our model were literature-derived from Patterson et al., 2019 rather than trial-derived. Patterson et al. reported utilities directly using the EQ-5D instrument; therefore, the utility values were applied directly in our model and no mapping was required. Utilities were assigned at the health-state level in the partitioned survival model (progression-free survival [PFS] and progressive disease [PD]) and assumed constant within each health state. The utility values used for each health state, together with their probability distributions for probabilistic sensitivity analysis and full citations, are reported in Table 1.

We also clarified the handling of adverse-event (AE) disutilities. Disutilities were applied as temporary utility decrements with a one-cycle duration (21 days). Consistent with prior aUC economic evaluations, we applied a disutility of 0.01 for grade 1–2 AEs and 0.28 for grade ≥3 AEs. These decrements were weighted by treatment-arm AE incidence reported in EV-302. The timing/duration assumption and the application approach are documented here, and the corresponding parameter values, distributions, and sources are provided in Table 1.
