# Supplementary material for: Cost-effectiveness analysis of Enfortumab vedotin and pembrolizumab for advanced urothelial carcinoma
Source: Medicine (Baltimore). 2026 Apr 10;105(15):e48312. doi: 10.1097/MD.0000000000048312 (PMC13593259; doi:10.1097/MD.0000000000048312)
Supplement: Supplementary file 2 [file medi-105-e48312-s002.pdf]

Table S1 Goodness-of-fit results for Overall Survival of Enfortumab vedotin and pembrolizumab

Model selection: Log-logistic model; Reason: Smallest AIC

Model	Ln-likelihood	Number of Parameters	AIC Value
llogis	-72.45552348	2	148.911047
RP-odds-1	-72.45576122	2	148.9115224
FP1-1	-72.46585091	2	148.9317018
gamma	-72.52533918	2	149.0506784
weibull	-72.58827243	2	149.1765449
RP-hazard-1	-72.58827251	2	149.176545
FP1-2	-72.83409221	2	149.6681844
RCS1	-72.08788442	3	150.1757688
FP2-1	-72.31929021	3	150.6385804
RP-normal-1	-72.32875368	3	150.6575074
RP-odds-2	-72.36131053	3	150.7226211
FP2-2	-72.36448654	3	150.7289731
gompertz	-73.47779724	2	150.9555945
gengamma	-72.53651142	3	151.0730228
exp	-74.54112039	1	151.0822408
RP-hazard-2	-72.59292182	3	151.1858436
RCS2	-71.99038809	4	151.9807762
genf	-72.25474088	4	152.5094818
RP-normal-2	-72.3345695	4	152.669139
lnorm	-74.77205349	2	153.544107

Table S2 Goodness-of-fit results for Overall Survival of Chemotherapy

Model selection: RP-odds model; Reason: Smallest AIC

Model	Ln-likelihood	Number of Parameters	AIC Value
RP-odds-1	-70.67013298	2	145.340266
llogis	-70.6701346	2	145.3402692
FP1-1	-71.63169208	2	147.2633842
RP-odds-2	-70.643136	3	147.286272
gamma	-71.64349318	2	147.2869864
FP2-1	-70.68945877	3	147.3789175
RP-normal-1	-70.86472049	3	147.729441
RCS2	-69.95846468	4	147.9169294
FP2-2	-71.12444456	3	148.2488891
gengamma	-71.23823311	3	148.4764662
RP-hazard-1	-71.37424794	3	148.7484959
RP-normal-2	-70.39371615	4	148.7874323
RP-hazard-2	-72.47109886	2	148.9421977
weibull	-72.47109944	2	148.9421989
genf	-70.61039624	4	149.2207925
RCS1	-71.83098582	3	149.6619716
FP1-2	-73.54776388	2	151.0955278
lnorm	-75.10467595	2	154.2093519
gompertz	-77.17048795	2	158.3409759
exp	-81.62834443	1	165.2566889

Table S3 Goodness-of-fit results for Progression-free Survival of Enfortumab vedotin and pembrolizumab

Model selection: RP-hazard model; Reason: Smallest AIC.

Model	Ln-likelihood	Number of Parameters	AIC Value
RP-hazard-1	-89.72098526	4	187.4419705
RP-odds-1	-89.85718416	4	187.7143683
RP-normal-1	-89.86998463	4	187.7399693
FP2-1	-91.34660674	3	188.6932135
genf	-91.14808931	4	190.2961786
FP2-2	-92.47709243	3	190.9541849
RP-hazard-2	-89.50288325	6	191.0057665
RP-odds-2	-89.52042595	6	191.0408519
RP-normal-2	-89.85508471	6	191.7101694
RCS2	-91.90960374	4	191.8192075
gengamma	-95.02337498	3	196.04675
lnorm	-96.11103547	2	196.2220709
RCS1	-97.85991975	3	201.7198395
llogis	-100.1218501	2	204.2437002
gompertz	-105.5497359	2	215.0994718
FP1-2	-106.3885262	2	216.7770525
exp	-108.8346334	1	219.6692669
gamma	-108.589128	2	221.178256
FP1-1	-108.6417634	2	221.2835268
weibull	-108.9149699	2	221.8299398

Table S4 Goodness-of-fit results for Progression-free Survival of Chemotherapy

Model selection: RP-odds model; Reason: Smallest AIC

Model	Ln-likelihood	Number of Parameters	AIC Value
RP-odds-2	-90.17722723	5	190.3544545
RP-normal-2	-91.59981484	5	193.1996297
RP-hazard-1	-90.14669166	7	194.2933833
RP-odds-1	-90.58135417	7	195.1627083
RP-normal-1	-91.51110466	7	197.0222093
RP-hazard-2	-93.04426243	6	198.0885249
llogis	-99.90631789	2	203.8126358
FP2-1	-101.4474683	3	208.8949366
lnorm	-103.9125516	2	211.8251033
gengamma	-103.1686776	3	212.3373553
FP2-2	-103.5299522	3	213.0599044
RCS2	-103.8356515	4	215.671303
RCS1	-107.6741314	3	221.3482628
FP1-2	-109.787395	2	223.57479
gamma	-110.6098888	2	225.2197775
FP1-1	-112.2636229	2	228.5272458
weibull	-117.7893498	2	239.5786996
gompertz	-134.7777558	2	273.5555117
exp	-138.8718621	1	279.7437241
RP-odds-2	-90.17722723	5	190.3544545

Table S5 Breakdown of Disease Management Costs

**Resource**	**No. required per week**	**Source (resource use)**	**Unit cost, $**	**Source (unit cost)**
GP consultation	0.23	NICE TA49210	131.89	Centers for Medicaid and Medicare Services (CMS), Physician Fee Schedule
Community nurse visit	0.92		93.2	
Health home	0.23		131.66	
Dietician	0.23		93.2	
Oncology	0.23		93.2	
	**Total cost per week ($):**		189.22	

Table S6 Breakdown of Administration Cost per Included Treatment

Type of administration	Cost per administration, $	Source/comment
Chemotherapy IV infusion (1 hour)—non-facility price, national payment amount	132.16	Centers for Medicaid and Medicare Services (CMS), Physician Fee Schedule

Table S7 Breakdown of Monitoring Costs per Included Treatment

Monitoring cost	Frequency per week	Source	Unit cost, $	Source
Outpatient visit	0.23	FDA label – Emc13	31.18	Centers for Medicaid and Medicare Services (CMS), Physician Fee Schedule
Hepatic function test	0.23	FDA label – Emc13	8.17	Centers for Medicaid and Medicare Services (CMS), Physician Fee Schedule
Renal function	0.23	FDA label – Emc13	8.68	Centers for Medicaid and Medicare Services (CMS), Physician Fee Schedule
Thyroid test	0.23	FDA label – Emc13	16.8	Centers for Medicaid and Medicare Services (CMS), Physician Fee Schedule
**Total cost per week ($)**			14.9	
