# Supplementary material for: Cost-effectiveness analysis of Enfortumab vedotin and pembrolizumab for advanced urothelial carcinoma
Source: Medicine (Baltimore). 2026 Apr 10;105(15):e48312. doi: 10.1097/MD.0000000000048312 (PMC13593259; doi:10.1097/MD.0000000000048312)
Supplement: Supplementary file 3 [file medi-105-e48312-s003.pdf]

Figure S1 Visual Goodness-of-fit results for Overall Survival of Enfortumab vedotin and pembrolizumab

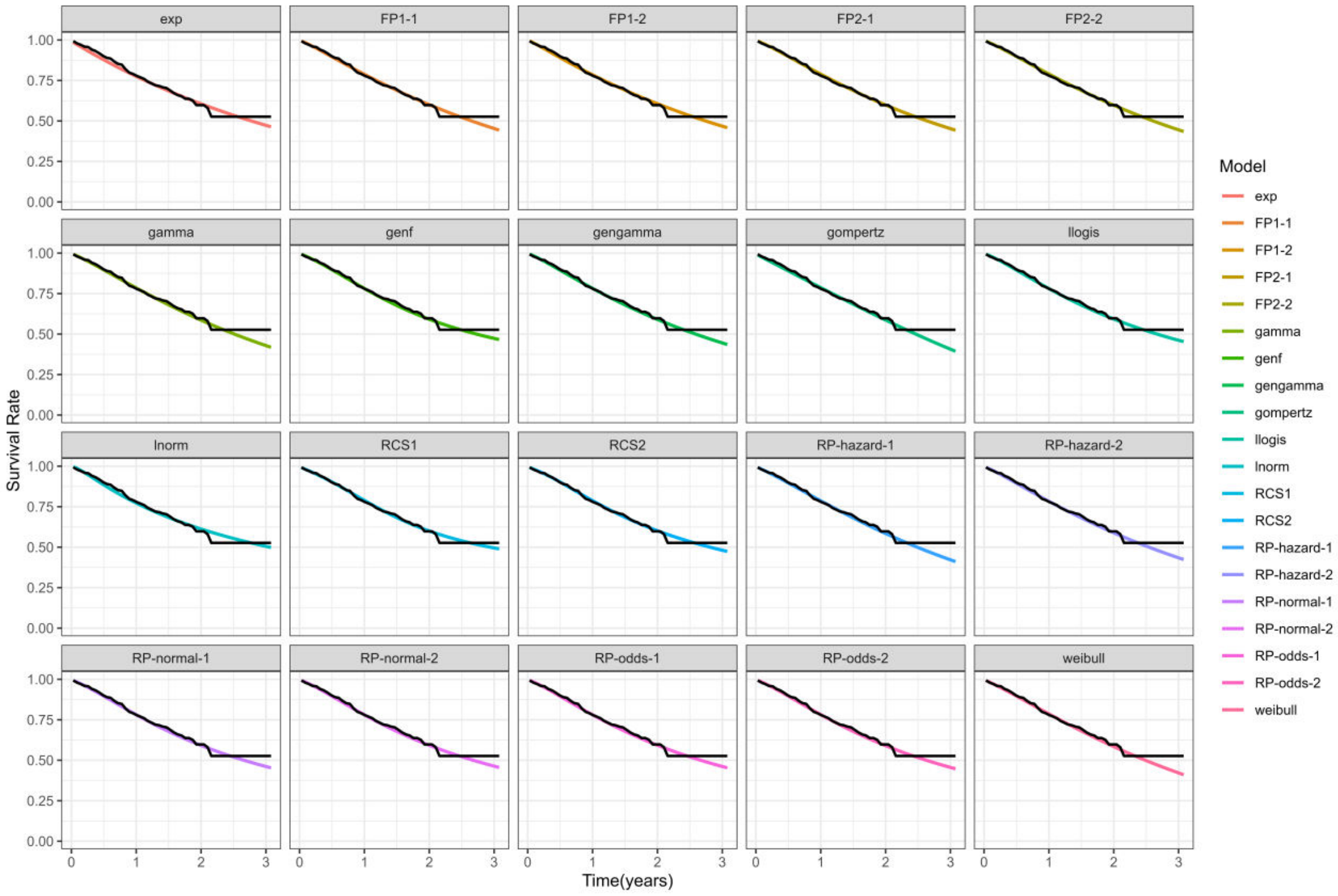

Figure S2 Visual Goodness-of-fit results for Overall Survival of Chemotherapy

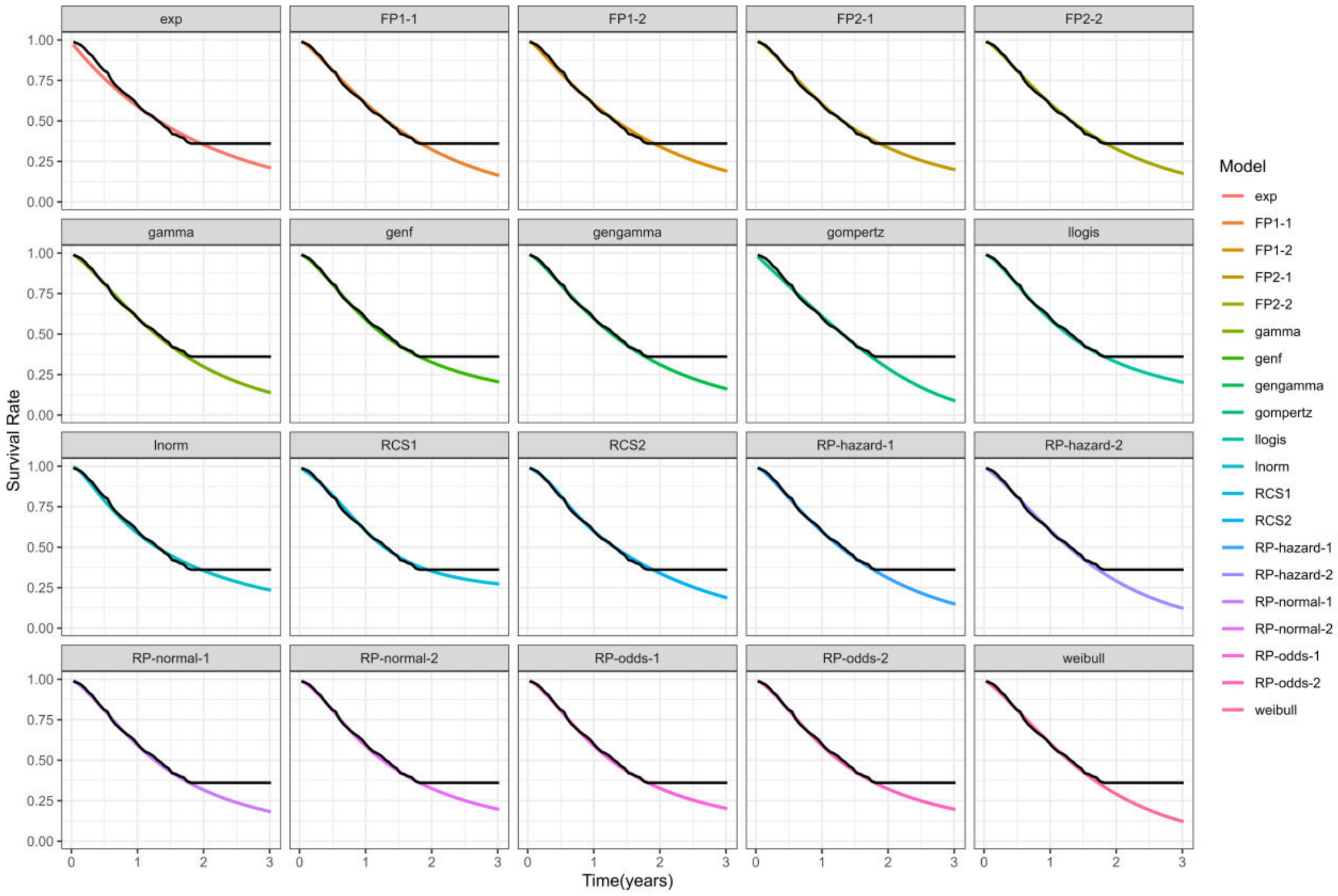

Figure S3 Visual Goodness-of-fit results for Progression-free Survival of Enfortumab vedotin and pembrolizumab

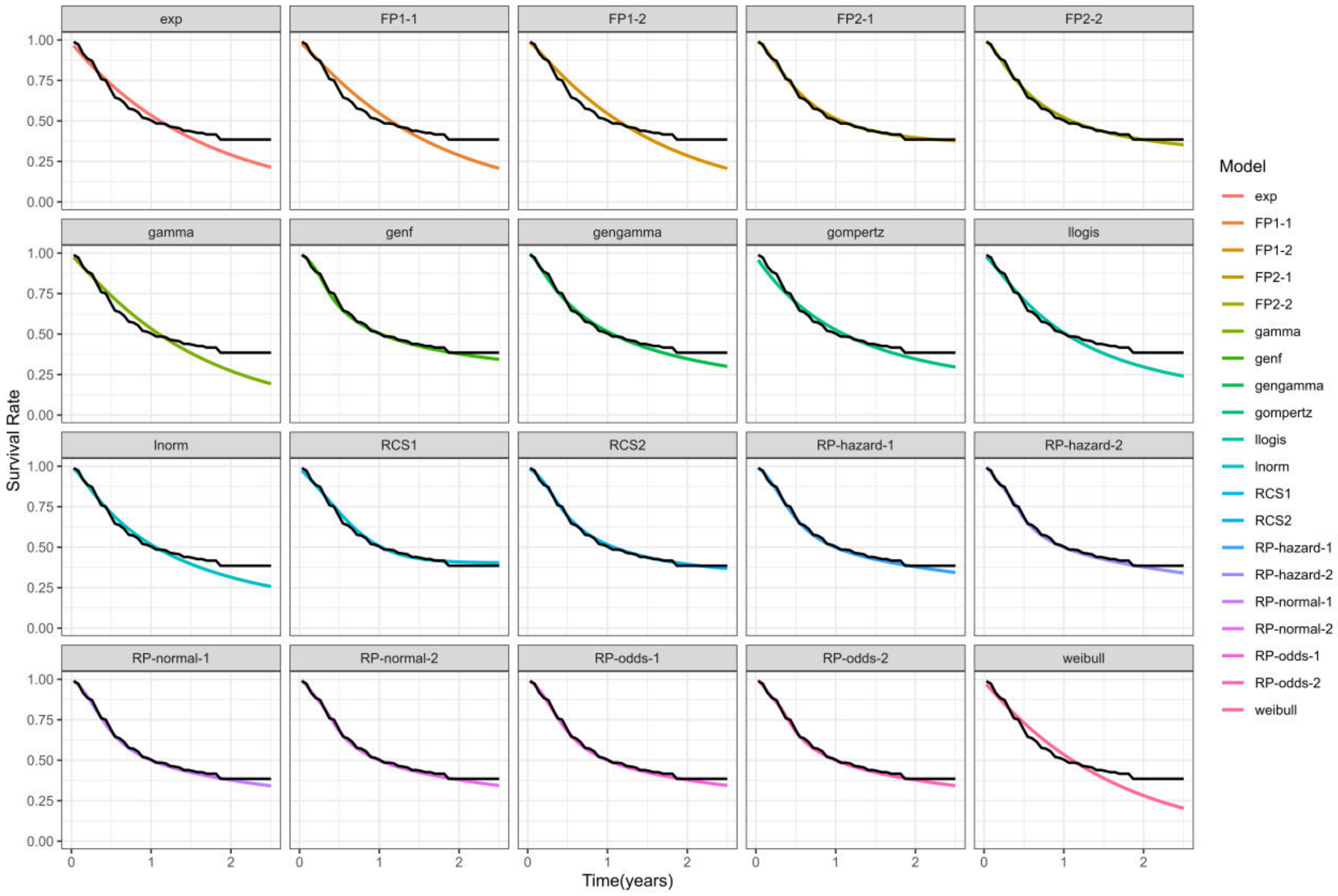

Figure S4 Visual Goodness-of-fit results for Progression-free Survival of Chemotherapy

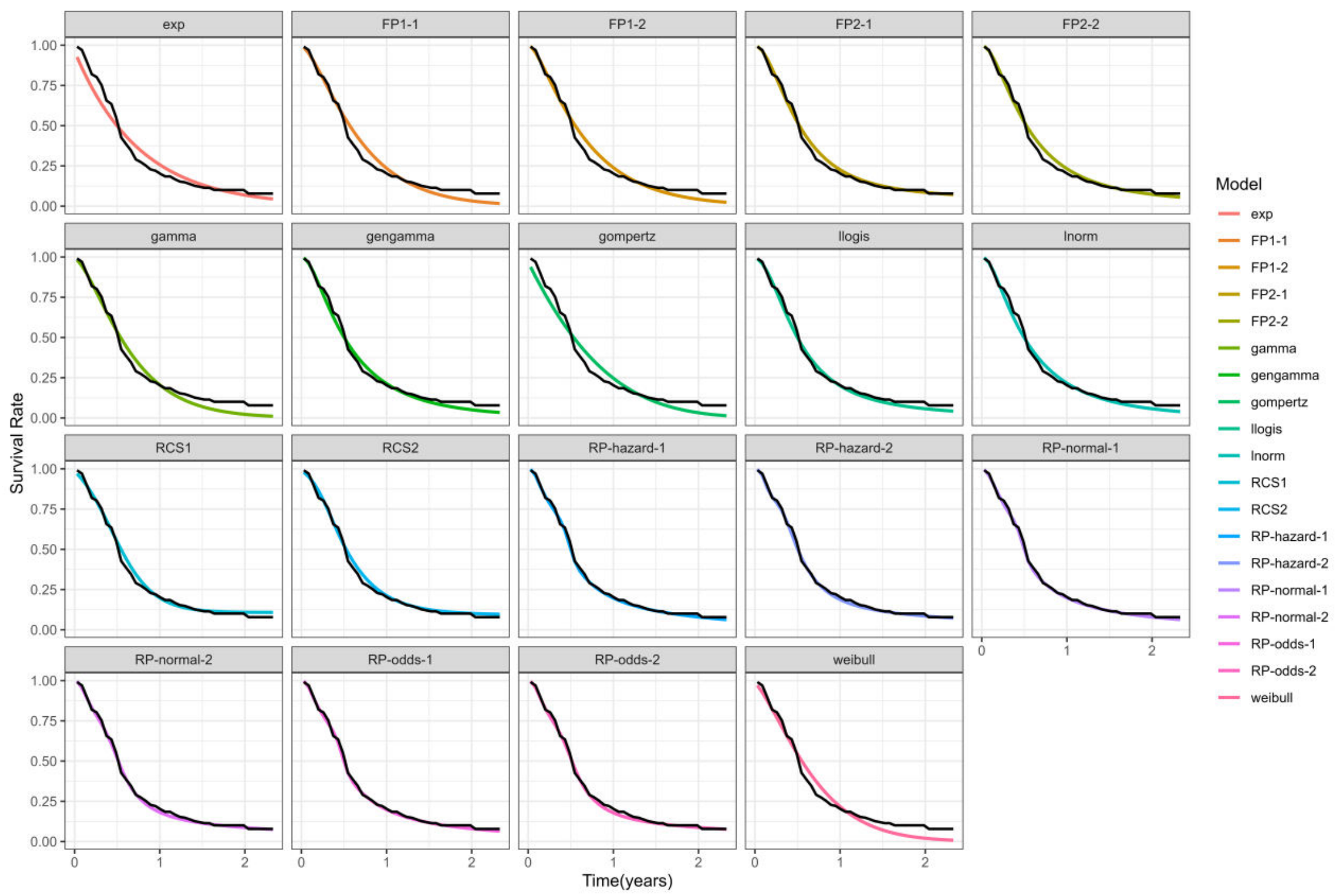

Figure S5 Summary of Subsequent Therapy in EV-302

**Parameters**	**Enfortumab vedotin–pembrolizumab (N=442)**	**Chemotherapy (N=444)**
Number of patients (percent)		
Patients who remained on treatment	144 (32.6)	0
Patients who received subsequent anticancer therapies	140 (31.7)	313 (70.5)
First subsequent systemic therapy	128 (29.0)	294 (66.2)
Platinum-based therapy	110 (24.9)	17 (3.8)
PD-1/PD-L1 inhibitor-containing therapy	7 (1.6)	260 (58.6)
Maintenance therapy*,†	0	143 (32.2)
Avelumab	0	135 (30.4)
Other therapy	7 (1.6)	117 (26.4)

*Included atezolizumab, avelumab, ipilimumab, M 6223, nivolumab, Nktr 255, and pembrolizumab.
†Maintenance therapy was permitted in the trial after platinum-based chemotherapy.
PD-1, programmed cell death protein 1; PD-L1, programmed death-ligand 1.

Figure S6 Additional Progression-Free Survival information

Subgroup	Enfortumab Vedotin–Pembrolizumab	Chemotherapy
	mo (no. of events/no. of patients)	
Overall	12.5 (223/442)	6.3 (307/444)
Age		
<65 yr	12.7 (75/144)	6.4 (88/135)
≥65 yr	12.0 (148/298)	6.2 (219/309)
Race		
White	10.4 (168/308)	6.2 (207/290)
Other	22.3 (55/134)	6.5 (100/154)
Geographic region		
North America	12.0 (58/103)	6.3 (55/85)
Europe	10.4 (94/172)	6.3 (144/197)
Rest of the world	NE (71/167)	6.2 (108/162)
Sex		
Female	10.4 (55/98)	6.1 (74/108)
Male	14.6 (168/344)	6.3 (233/336)
ECOG performance-status score		
0	22.3 (93/223)	6.7 (146/215)
1 or 2	9.3 (130/219)	6.1 (161/227)
Primary site of origin of disease		
Upper tract	12.7 (69/135)	6.2 (70/104)
Lower tract	12.5 (152/305)	6.3 (236/339)
Liver metastases		
Present	8.2 (66/100)	6.0 (78/99)
Absent	16.4 (157/342)	6.4 (229/345)
PD-L1 expression		
Low (CPS <10)	10.5 (105/184)	6.3 (127/185)
High (CPS ≥10)	18.5 (116/254)	6.2 (176/254)
Cisplatin eligibility status		
Eligible	14.6 (117/244)	6.5 (149/234)
Ineligible	10.6 (106/198)	6.1 (158/210)
Site of metastasis		
Visceral site	10.4 (176/318)	6.2 (238/318)
Lymph node only	NE (38/103)	8.3 (55/104)
Renal function		
Normal	18.7 (38/84)	6.7 (61/95)
Mild impairment	12.7 (79/165)	6.3 (114/162)
Moderate or severe impairment	10.5 (106/193)	6.2 (132/187)

Note, from N Engl J Med 2024;390:875-88

Figure S7 Additional Overall Survival information

Subgroup	Enfortumab Vedotin–Pembrolizumab	Chemotherapy
	mo (no. of events/no. of patients)	
Overall	31.5 (133/442)	16.1 (226/444)
Age		
<65 yr	NE (39/144)	19.7 (58/135)
≥65 yr	31.5 (94/298)	14.6 (168/309)
Race		
White	26.1 (104/308)	15.3 (162/290)
Other	NE (29/134)	19.3 (64/154)
Geographic region		
North America	25.6 (40/103)	21.2 (42/85)
Europe	NE (56/172)	13.9 (110/197)
Rest of the world	NE (37/167)	16.4 (74/162)
Sex		
Female	25.4 (32/98)	14.6 (54/108)
Male	31.5 (101/344)	16.6 (172/336)
ECOG performance-status score		
0	NE (44/223)	18.4 (94/215)
1 or 2	25.4 (89/219)	13.1 (131/227)
Primary site of origin of disease		
Upper tract	NE (38/135)	18.4 (45/104)
Lower tract	31.5 (94/305)	15.6 (180/339)
Liver metastases		
Present	19.1 (43/100)	10.1 (67/99)
Absent	NE (90/342)	17.9 (159/345)
PD-L1 expression		
Low (CPS <10)	NE (53/184)	15.5 (99/185)
High (CPS ≥10)	31.5 (79/254)	16.6 (125/254)
Cisplatin eligibility status		
Eligible	31.5 (69/244)	18.4 (106/234)
Ineligible	NE (64/198)	12.7 (120/210)
Site of metastasis		
Visceral site	25.6 (108/318)	13.6 (182/318)
Lymph node only	NE (22/103)	27.5 (39/104)
Renal function		
Normal	26.1 (24/84)	18.4 (44/95)
Mild impairment	NE (42/165)	16.4 (78/162)
Moderate or severe impairment	31.5 (67/193)	13.3 (104/187)

Note, from N Engl J Med 2024;390:875-88
